# Lysogeny of Escherichia coli by the Obligately Lytic Bacteriophage T1: Not Proven

**DOI:** 10.1128/mBio.00740-21

**Published:** 2021-05-04

**Authors:** Michael G. Jobling

**Affiliations:** a Retired Faculty, Department of Immunology and Microbiology, University of Colorado School of Medicine, Aurora, Colorado, USA; Johns Hopkins Bloomberg School of Public Health

**Keywords:** bacteriophages, lysogeny, phage T1

## LETTER

The work by Laganenka et al. (1) is to be welcomed as a rare molecular study on a close relative of the classical lytic T1 bacteriophage (2, 3). Unfortunately, the evidence for lysogeny is weak, and further work should have been done in support of the proposed lysogenic state. Several aspects of this work are concerning and point to unintended contamination as an explanation for the proposed “lysogenic” state.

First, and most problematic, the genome assembly for their lysogen contains not even a partial T1 contig. On this, the authors were silent. The section covering the genome sequence implies otherwise, suggesting that the phage genome is found extrachromosomally, with no flanking host chromosomal sequences. The latter statement applies only to purified phage sequenced from a lytic culture. That the authors did not find this troubling is troubling in and of itself. The putative lysogen cannot be both extremely stable (100% positivity by PCR after 28-fold single colony purification of 10 replicates; their Fig. S2A) and yet not present in a genomic DNA preparation. Absent evidence to the contrary, phage and host genomes should be equally represented in a sequencing library generated from a stable lysogen, with at least one phage genome per host chromosome. Yet more than 1.6 million paired reads from the SRA data file (https://www.ebi.ac.uk/ena/browser/view/SRR8695865) map to the host chromosome, but only a single read pair maps to the T1 genome. This is indicative of low-level contamination, not lysogeny. When asked, the authors suggested low-efficiency DNA purification for the phage genome, low proportions of unintegrated phage DNA present, or low quality of phage DNA as unlikely reasons for producing no meaningful sequence reads for the T1 genome. All of these have major implications for the lysogeny claim, and none of these explanations are compatible with lysogen stability. The ATCC has recently and independently produced a closed circular genome for this strain, with no T1 sequences (https://genomes.atcc.org/genomes?text=15144). This must be addressed.

Second, stable lysogeny is inexplicably suggested to be specific for ATCC 15144, a wild-type B strain. Colonies appearing in phage plaques on other wild-type ECOR isolates are suggested to be “unstable lysogens,” isolated within clear plaques of T1-infected isolates (their Fig. S2C), which then failed to detect T1 DNA by PCR after streak-purifying and regrowth. It is possible that these survivors are T1^R^ mutants (*fhuA*/*tonB*?) and not unstable lysogens; picking directly from the plaque into broth will inevitably transfer free phage particles, detected by PCR; restreaking should separate free phage from T1^R^ cells, which then would test negative by PCR for T1. It does not appear that the ability of T1 to plaque on these derivatives of ECOR strains was tested, or if unstable lysogens could be repeatedly generated from surviving colonies.

Third, although the inability of the phage to infect another B strain, BL21(DE3), is presented as a conundrum, that strain may actually be T1^R^. Several commercial sources of BL21(DE3), e.g., NEB (E. Raleigh, personal communication) are supplied [still using the BL21(DE3) designation] as *fhuA*2 derivatives (and therefore T1^R^) of the original strain (4); *fhuA*2 is an IS*2* insert originally isolated as *tonA*2, the gene encoding the receptor used by T1 phage. The original source of the lab stock should be clarified.

Fourth, no PFU were ever detected in nonlysed cultures (their Fig. S1 legend), a level of repression not found for any other nondefective lysogenic phage, and any phage replication in a culture (even putative lysogens) led to complete lysis, indicative of truly lytic infections.

A concurrent paper from another group (5) proposed that a different T1-like phage also deviates from the obligately lytic lifestyle but specifically ruled out lysogeny; they reported it forms a loosely defined nonlysogenic carrier state, benefiting the host strain. The lytic phage was reportedly “carried” by the host strain (viable phage were also not detectable), but they could also not detect the phage genome in these carriers, proposing that the phage exists extracellularly. How this would prevent detection is not explained. A low level of PFU could be detected only if a gene from a different host prophage was overexpressed. These T1-like phage could then plaque on the carrier host strain and were also lytic for the phage-less host. Both of these works could be documenting a similar property of T1-like phage, likely persistent contamination, a documented phenomenon in bacterial artificial chromosome (BAC) libraries (cited in references 6 and 7); it is problematic on both an industrial scale (8, 9) and anecdotally at the research level (10). Persistence may be aided by T1s’ (and phi80s’) requirement for metabolically active cells for infection (11). In this work I conclude that its detection has prematurely, from the data presented, been attributed to lysogeny.
